# *Rhodomyrtus tomentosa* Fruits in Two Ripening Stages: Chemical Compositions, Antioxidant Capacity and Digestive Enzymes Inhibitory Activity

**DOI:** 10.3390/antiox11071390

**Published:** 2022-07-18

**Authors:** Xiaoping Hu, Yuting Chen, Jincheng Dai, Linling Yao, Lu Wang

**Affiliations:** 1 Key Laboratory of Food Nutrition and Functional Food of Hainan Province, College of Food Science and Engineering, Hainan University, Haikou 570228, China; huxiaoping03@hainanu.edu.cn (X.H.); 20190881310055@hainanu.edu.cn (Y.C.); 20203103943@hainanu.edu.cn (J.D.); 19095135210020@hainanu.edu.cn (L.Y.); 2 Engineering Research Center of Utilization of Tropical Polysaccharide Resources, Ministry of Education, Hainan University, Haikou 570228, China

**Keywords:** *Rhodomyrtus tomentosa* fruit, nutritional characteristics, phenolic compositions, anti-oxidant activity, digestive enzymes inhibitory activity, molecular docking analysis

## Abstract

*Rhodomyrtus tomentosa* fruit (RTF) has been known as a food source with multiple health-care components. In this work, nutrition characteristics, free and bound phenolic profiles, antioxidant properties in vitro and digestive enzymes inhibitory activities of un-fully mature RTF (UM-RTF) and fully mature RTF (FM-RTF) were evaluated for the first time. Results verified that high levels of energy, ascorbic acid, organic acids and total phenolics were observed in FM-RTF. Moreover, FM-RTF had significant higher total phenolic content (TPC), but significantly lower total flavonoid content (TFC) than UM-RTF. In addition, twenty phenolic compounds in RTF were identified by high performance liquid chromatography–electrospray ionization–quadrupole time-of-flight tandem mass spectrometry (HPLC-ESI-qTOF-MS/MS) method. Quantitative analysis results indicated that gallic acid, ellagic acid and astragalin were the predominant free phenolics, while gallic acid and syringetin-3-*O*-glucoside were dominant in bound phenolic fractions. In contrast, higher contents of phenolics were observed in FM-RTF. The results also confirmed that FM-RTF exhibited higher antioxidant activities and digestive enzymes inhibitory activities than UM-RTF. Strong inhibitory ability on α-glucosidase was found in RTF, while bound phenolics showed a stronger α-amylase inhibitory effect than free phenolics. Moreover, the interaction between the main phenolic compounds and α-glucosidase/α-amylase was preliminary explored by molecular docking analysis. The results provided valuable data about the chemical compositions and biological potential of *R. tomentosa* fruits in both maturation stages studied.

## 1. Introduction

*Rhodomyrtus tomentosa* (Ait.) Hassk is widely cultivated in southern China, Japan, India, Philippines, Malaysia, Vietnam and Indonesia [1,2]. As a ‘‘Neglected and Underutilised Crop Specie’’, this plant has been considered as traditional folk medicine for a long time [1,2,3]. *Rhodomyrtus tomentosa* fruit (RTF) consists of various nutrients, including phenolic acids, anthocyanins, flavonoids, organic acids and amino acids [3,4]. Many researchers have verified that RTF has multiplex health-promoting benefits, including anti-oxidant, anti-inflammatory, anti-hypoglycemic, anti-diarrheal, anti-obesity and anti-non-alcoholic fatty liver effects [4,5,6].

It has been found that the ripeness of fruits has a great influence on the nutritional characteristics, chemical components and biological properties [7,8]. Normally, total soluble solids, amino acids, sugars, phenolics, anthocyanins and bio-activities in berry fruits tend to increase during ripening [9,10,11]. In the folk medicine, un-fully mature RTF (UM-RTF) is generally used to treat dysentery or diarrhea. In contrast, fully mature RTF (FM-RTF), is considered as a diet source rich in natural antioxidants [5,6,10]. Hence, understanding the changes in chemical compositions is of great importance for determining the best ripening stage for fruits with better quality and nutritional value [12,13,14]. Lai et al. (2013) reported that RTF extract consisted of phenolic acids, flavonols, anthocyanins, ellagitannins and stilbenes. Among them, piceatannol is a dominant phenolic compound [9]. Currently, studies on *R. tomentosa* are mainly concentrated on the phytochemicals from its leaves, flowers and stems owing to their antioxidant, anti-bacterial, anti-inflammatory properties and DNA damage prevention effects [15,16,17,18]. A comprehensive analysis of the nutritional characteristics, chemical composition and bio-activities of RTF during the ripening stage has not yet been carried out.

Taking into account the medicinal value of this edible fruit, this study aims to analyze nutritional compositions, free and bound phenolic compounds of RTF in two ripening stages. Additionally, the antioxidant activities in vitro and digestive enzymes inhibitory ability of free and bound phenolic fractions of UM-RTF and FM-RTF were also investigated. More importantly, the digestive enzyme inhibitory activities of the main phenolic compounds were revealed by molecular docking analysis. This preliminary study may highlight the potential valuable of this unexploited fruit, thus promoting its development and application in the food industry.

## 2. Materials and Methods

### 2.1. Materials and Chemicals

*R. ulmifolius* fruits were collected from a planting base in Chengmai, Hainan, China (110.01″ E, 19.80″ N). Un-fully mature *R. ulmifolius* fruits (UM-RTF) were harvested on July, 01, 2021, which were a month earlier than fully mature *R. ulmifolius* fruits (FM-RTF). The samples (UM-RTF and FM-RTF) were first freeze-dried, and then ground into fine powder, before being sifted using a 40-mesh sieve. After that, the samples were stored at a refrigerator (4 °C) for subsequent experiments. Folin–Ciocalteu reagent, 1,1-diphenyl-2-picrylhydrazyl (DPPH), 2,2′-azinobis(3-ethylbenzothiazoline-6-sulfonic acid ammonium salt (ABTS), 2,4,6-tris(2-pyridil)-s-triazine (TPTZ) and 6-hydroxy-2,5,7,8-tetramethylchroman-2-carboxylic acid (Trolox) were purchased from Aladdin (Shanghai, China). α-Glucosidase from *Saccharomyces cerevisiae* (≥10 units/mg protein); α-amylase from porcine pancreas (≥5 units/mg solid); HPLC-grade of phenolic standards, including gallic acid, protocatechuic acid, vanillic acid, *p*-hydroxycinnamic acid, ellagic acid, luteolin-7-*O*-glucoside, astragalin, syringetin-3-*O*-glucoside and naringenin (>98%); monosaccharide standards (>98%); organic acids standards (>99%) and amino acid standards (>99%) were purchased from Sigma-Aldrich (St. Louis, MO, USA). Other analytical-grade chemicals were brought from Damao Chemical Reagent Co., Ltd. (Tianjin, China). Ultra-pure water was prepared by Milli-Q ultra-pure apparatus (Millipore, Bedford, MA, USA).

### 2.2. Nutritional Assessment

The AOAC method was adopted to determine the contents of protein, carbohydrates and ash of UM-RTF and FM-RTF [19]. The calculation of total energy was implemented according to the formula: Energy (kcal) = 4 × (g protein + g carbohydrates) + 9 × (g fat) [20].

Free sugars of UM-RTF and FM-RTF were extracted by the procedure described in [19]. The contents of free sugars of UM-RTF and FM-RTF were determined by a 1260 HPLC system equipped with a refraction index detector. Prior to HPLC analysis, the extracts were filtered using 0.45 μm Whatman nylon filters. The free sugars were quantified by the internal standard method and the results were expressed as mg/g DW [21].

The contents of organic acids of UM-RTF and FM-RTF were measured using a 1260 HPLC system coupled with a photodiode array detector by using the method reported by Pereira et al. [22]. The free organic acids were quantified by the internal standard method and results were expressed as μg/g DW.

The contents of free amino acids of UM-RTF and FM-RTF were analyzed by the method by Song et al. [23]. The separation of the free amino acids of the extracts was conducted using an L-8900 automatic amino acid analyzer (Hitachi Co. Ltd., Tokyo, Japan), equipped with a 2622 Hitachi custom ion-exchange resin column (60 × 4.6 mm, 5 μm, Tokyo, Japan). Briefly, the samples (1.0 g) were extracted with 20 mL 1% sulfo salicylic acid under ultrasonic powder of 320 W for 30 min. The chromatographic conditions were in agreement with the methods described by Song et al. [23]. The contents of free amino acids were calculated by the internal standards method, and results were expressed as mg/g DW.

### 2.3. Extraction of Free and Bound Phenolic Fractions

Different phenolic fractions were obtained according to the method of Wang et al. 2019 [24]. Figure S1 showed the flow diagram of extraction and analysis for UM-RTF and FM-RTF. Briefly, RTF powder (2 g) was mixed with 10 mL of 70% ethanol/water (*v*/*v*) in a 15-mL Eppendorf tube, followed by extraction twice in an ultrasonic bath of 320 W at 50 °C for 30 min. After that, centrifugal treatment was conducted at 5000× *g* for 10 min at 4 °C, and the combined filtrate was evaporated to dryness in vacuum at 30 °C. The dryness was resolved in 5 mL of 50% ethanol/water (*v*/*v*) to obtain free phenolic fractions. After free phenolic extraction, the residues were dried to a fixed weight at 50 °C, then used to extract the bound phenolics. The above residue (1 g) was soaked in 40 mL of 2 M NaOH at 30 °C for 2 h in nitrogen. After that, pH value of the hydrolysate was adjusted to 2 by using 6 M HCl. The mixture was degreased three times with 50 mL hexane. Then, the supernatant was extracted three times with 15 mL of diethyl ether/ethyl acetate (1:1, *v*/*v*) using the method by Wang et al. [24]. The combined extraction was evaporated to dryness in vacuum at 30 °C. The dryness was reconstituted in 5 mL of 50% ethanol to obtain bound phenolic fractions. Free and bound phenolic fractions were stored at −20 °C for later use.

### 2.4. Measurement of Phenolic and Flavonoid Contents

The determination of phenolic content in free and bound phenolic fractions was carried out using the Folin–Ciocalteau method by Wu et al. [25]. Gallic acid with concentration ranging from 0.1–1.0 mg/mL was used as the standard. Results were expressed in mg gallic acid equivalents per g sample in dry weight (mg GAE/g DW). The content of free and bound flavonoid was measured using the reported method of Li et al. [26] by taking rutin as the standard. Results were expressed in mg rutin equivalents per g sample in dry weight (mg RE/g DW).

### 2.5. HPLC-ESI-qTOF/MS and HPLC-DAD Analysis

The phenolic compositions were identified using an Agilent 1260 HPLC system coupled with a high-resolution time-of-flight (HR-qTOF) mass detector and an electrospray ionization (ESI) source. An Aligent Zorbax Eclipse C18 plus column was adopted to identify the compounds. Two mobile phases consisted of acetonitrile-0.1% formic acid (A) and water-formic acid (B). The gradient elution program was as follows: 0–5 min, 15% A; 5–30 min, 15–35% A; 30–40 min, 35–50% A; 40–45 min, 80% A; 45–50 min, 15% A at a flow rate of 0.8 mL/min. The other chromatogram conditions were as follows: injection volume of 10 µL, column temperature of 30 °C;, and the detection wavelength ranging from 200 to 600 nm. The ESI source conditions were referred to our previously described method [27,28]. Bruker compass DataAnalysis software was used to acquire MS data. The identified compounds were quantified using HPLC-DAD method, and the chromatographic conditions were consistent with the above developed HPLC-ESI-qTOF-MS/MS. The contents of the main compounds were expressed in μg/g DW.

### 2.6. Antioxidant Activity

The DPPH and ABTS^+^ assays were conducted following the previous protocols by Wang et al. [24]. The scavenging results of DPPH and ABTS^+^ were expressed as micromole trolox equivalents per gram dried weight (μmol TE/g DW). The determination of OH^−^ scavenging ability was performed using the method proposed by Wang et al. [24], and the result were expressed in μmol TE/g DW as well. The FRAP assay was conducted following the same method in [29]. The FRAP value was expressed as micromoles of ferrous sulfate equivalents (Fe(II)SE) per gram of dried weight (μM Fe(II)SE/g DW).

### 2.7. Digestive Enzyme Inhibition Activities

#### 2.7.1. α-Glucosidase Inhibitory Activity

The α-glucosidase inhibitory activity (α-GIA) of the extracts was measured using the method by Li et al. [26]. In short, 1.0 U/mL α-glucosidase (50 μL) dissolving in 0.01 M PBS (pH 6.9), 50 μL of the diluted sample extracts (5, 10, 15, 20, 30, 60, 80, 120 μg/mL dissolving in 20% ethanol) and 100 μL of 0.01 M PBS (pH 6.9) were added to an Eppendorf tube and placed in a water bath for 10 min at 37 °C, followed by addition of 5.0 mM *p*-NPG solution (100 μL) for another 20 min of incubation at 37 °C. After that, 300 μL of 0.2 M Na_2_CO_3_ solution was added to terminate the reaction. Finally, the absorbance of the reaction was determined at 450 nm using a microplate reader (SpectraMax M5 Molecular Device, CA, USA). The α-glucosidase inhibition rate of the sample extracts was calculated by Equation (1):

(1) $$\mathrm{Inhibition}\;\mathrm{rate}\;(\%)\;=\;\left[1\;-\;\frac{\Delta A_{s}}{\Delta A_{c}\;}\right]\times 100\%$$

where ∆A_s_ = A_sample+enzyme_ − A_sample_, ∆A_c_ = A_PBS+enzyme_ − A_PBS_.

#### 2.7.2. α-Amylase Inhibition Activity

The determination of α-amylase inhibitory activity (α-AIA) was carried out using the method by Zhu et al. (2019) with slight modifications [30]. First, 100 μL of 0.5 U/mL α-amylase in PBS (pH = 6.9) was mixed thoroughly with 50 μL of the diluted sample extracts (1.5, 2.0, 2.5, 3.0, 3.5, 4.0, 5.0 mg/mL in 20% ethanol) or the phenolic standards, followed by incubation at 37 °C; for 10 min and addition of soluble starch solution (100 μL) for another 20 min of incubation at 37 °C;. After that, 200 μL of saturated Na_2_CO_3_ solution was added to terminate the reaction. Finally, the absorbance at 490 nm was determined by using a microplate reader (SpectraMax M5 Molecular Device, CA, USA). The α-amylase inhibition rate of the sample extracts was calculated by Equation (1).

### 2.8. Molecular Docking Analysis

The binding mechanisms between the major phenolic compounds and the receptors can be explained by molecular docking simulation method reported by Li et al. [26]. The 2D structure documents of the major phenolics compounds in NFE and acarbose were downloaded from the website (http://zinc.docking.org/, accessed on 12 July 2022). The 3D structures of α-glucosidase (PDB ID: 3A4A) and α-amylase (PDB ID: 1PPI) were acquired from the Protein Data Bank website (http://www.rcsb.org/pdb, accessed on 12 July 2022). Molecular docking in Surflex-Dock Geom (SFXC) mode were carried out using SYBYL-X 2.0 software. Before molecular docking analysis, α-glucosidase and α-amylase were treated by removing the ligands and water molecules and adding CHARMM force field and polar hydrogen. In order to obtain the optimal binding mode, the free energy minimization under the CHARMM force field was selected for docking. The docking parameters (C-score, T-score, interaction force types, hydrogen bonds distances and interaction sites) were gained from the molecular docking.

### 2.9. Statistic Analysis

All experimental tests were conducted in triplicate and the results were expressed as mean ± standard deviation. The experimental data were evaluated by Statistic software version 19.0. One-way analysis of variance (ANOVA) followed by Tukey’s HSD test and two-way ANOVA were conducted for significant difference analysis. The difference was considered significant when *p* < 0.05.

## 3. Results and Discussion

### 3.1. Nutritional Composition

Table 1 shows the nutritional composition of UM-RTF and FM-RTF. The statistical analysis verified that parameters for the UM-RTF and FM-RTF were significantly different (*p* ≤ 0.05). The FM-RTF showed the highest concentrations of moisture (32.43%), carbohydrates (65.29%), ascorbic acid (2.57 mg/g DW) and energy (81.23%), while UM-RTF exhibited the highest content of ash (19.87%). It could be observed that protein content of RTF was about 38.78–40.15 mg/g DW, which agreed with the result of Lai et al. [1]. The total organic acids content of FM-RTF (2273.84 μg/g DW) was significantly higher than that of UM-RTF (1707.64 μg/g DW). Seven organic acids were identified in RTF. The main organic acids measured in FM-RTF were malic acid (829.58 ± 7.62 μg/g DW), succinic acid (468.01 ± 43.35 μg/g DW), acetic acid (389.06 ± 30.57 μg/g DW), oxalic acid (335.14 ± 3.65 μg/g DW), pyruvic acid (211.92 ± 15.15 μg/g DW) and D-galacturonic acid (42.69 ± 3.61). The contents of most organic acids in FM-RTF were higher than those in UM-RTF. So far, there is a lack of primary data on the organic acids of RTF. Regarding the free sugar profiles, the main sugar found in the RTF was glucose (124.55 ± 1.79 mg/g DW for FM-RTF, 137.13 ± 4.96 mg/g DW for UM-RTF). No significant difference in total sugar contents was observed in two ripening stages of RTF. The amino acids content of RTF in two ripening stages was insignificantly different. The total amino acids content was in the range 15.27–16.21 mg/g DW. About 15 amino acids existed in RTF. Glutamic acid, L-arginine, tyrosine and leucine were the dominant amino acids of UM-RTF and FM-RTF, which were in line with the reports of Lai et al. (2015) [1]. Compared with the recommended daily intake, the RTF is a relatively good fruit source rich in amino acids [1,2]. FM-RTF presented a TPC (31.53 ± 1.36 mg GAE/g DW), which is lower than that in the study of Lai et al. [1] was significantly higher than that in the work of Huang et al. [31]. This may be caused by genetic variations of the samples. In addition, it can be observed that free phenolic content (12.43 mg GAE/g DW) and bound phenolic content (17.38 mg GAE/g DW) in FM-RTF were higher than those in UM-RTF. However, the opposite result was observed for TFC. UM-RTF had higher TFC (9.87 mg RE/g DW), free flavonoid content (5.32 mg RE/g DW) and bound flavonoid content (3.17 mg RE/g DW) than FM-RTF. Compared with other berry fruits, RTF is a better source rich in dietary polyphenols [1,2,31].

### 3.2. Identification and Quantification of Phenolic Compositions

**Figure 1:**
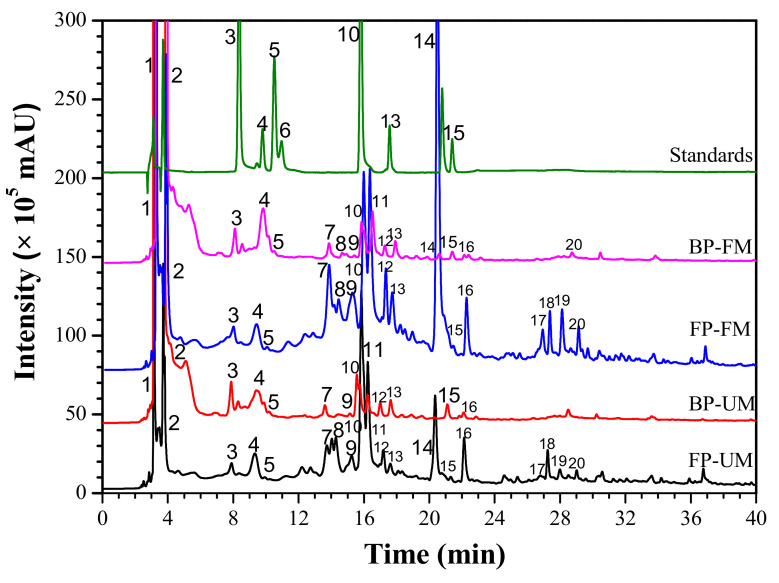
HPLC chromatogram (280 nm) of different extracts fractions of RTF in two ripening stages and the standards. FP-FM, free phenolic fraction of fully mature *R. tomentosa* fruits; FP-UM, free phenolic fraction of un-fully mature *R. tomentosa* fruits; BP-FM, bound phenolic fraction of fully mature *R. tomentosa* fruits; BP-UM, bound phenolic fraction of un-fully mature *R. tomentosa* fruits. Peaks: 1, gallic acid; 2, protocatechuic acid; 3, vanillic acid; 4, *p*-hydroxycinnamic acid; 5, ellagic acid; 10, luteolin-7-*O*-glucoside; 13, astragalin; 14, syringetin-3-*O*-glucoside; 15, naringenin.

The phenolic profiles of different fractions in two ripening stages were identified by HPLC-ESI-qTOF-MS/MS method (Figure 1). Table 2 presents the retention time, λ_max_, parent ion, main fragment ions and tentative identification. Seventy compounds were identified, consisting of eight phenolic acids (peaks 1–5, 11, 12 and 15), seven flavonoids/anthocyanins compounds (peaks 6, 7, 10, 13, 14, 18, and 20), two other compounds (Peaks 8 and 16) and three unknown compounds (peaks 9, 17 and 19).

Peak 1 (t_R_ = 3.015) with a [M + H]^+^ at *m*/*z* 170.02 was easily determined as gallic acid. Peak 2 (t_R_ = 3.672, [M + H]^+^ at *m*/*z* 153.02) was identified as protocatechuic acid. Peak 3 (t_R_ = 9.017) indicated the parent ion at m/z 169.15 [C_8_H_8_O_4_ + H]^+^, which can be easily identified as vanillic acid by comparison with reference standard. Peak 4 (t_R_ = 9.982) can be ascribed as *p*-hydroxycinnamic acid based on its parent ion *m*/*z* 165.14 [C_9_H_8_O_3_ + H]^+^. Ellagic acid (peak 5, t_R_ = 10.208, *m*/*z* 303.05 [C_14_H_6_O_8_ + H]^+^) was identified by comparing its retention time with the standard. Peak 6 (t_R_ = 13.727) presented adduct ions at *m*/*z* 463.12 [C_22_H_22_O_11_ + H]^+^. In MS^2^ spectra, it produced two main fragment ions at *m*/*z* 302.03 [C_22_H_22_O_11_ + H]^+^, *m*/*z* 162.10 [M-C_16_H_13_O_7_ + H]^+^ and *m*/*z* 161.05 [C_6_H_12_O_6_ + H]^+^ (loss of galactoside), which can be assumed as peonidin-3-*O*-*β*-galactoside by referring the reference [5,6]. Tricin 5-*O*-*β*-D-glucoside (peak 7, t_R_ = 14.259) was temporarily identified based on the parent ion [C_23_H_24_O_12_ + H]^+^ at *m*/*z* 493 and its fragment ions at *m*/*z* 332.29 [M-glc + H]^+^ and *m*/*z* 153.05 [-*O*-glc + H]^+^ (loss of glucoside)). Peak 8 was assumed as *p*-coumaraldehyde because of the adduct ion [C_9_H_8_O_2_ + H]^+^ at *m*/*z* 149.02 [5,6]. Peak 10, with the parent ion at *m*/*z* 449.10 [C_21_H_20_O_11_ + H]^+^ giving three main MS^2^ fragments ions *m*/*z* at 287.10 [C_15_H_10_O_6_ +H]^+^, 162.02 [M-C_15_H_10_O_7_ + H]^+^ and 153.02 [-O-glc + H]^+^, was determined as luteolin-7-*O*-glucoside. Peak 11 (t_R_ = 16.217, *m*/*z* 228.08 [C_14_H_12_O_3_ + H]^+^), indicating the MS^2^ fragment ions at *m*/*z* 185.06, 183.08, 159.08, 157.07 and 143.05, was temporarily confirmed as trans-resveratrol [32]. Based on the MS/MS fragmentation information and retention time, peak 12 (t_R_ = 17.195, *m*/*z* 195.05 [C_10_H_10_O_4_ + H]^+^) was determined as ferulic acid [27]. Peak 13 (t_R_ = 17.682), indicating a molecular ion at *m*/*z* 449.39 [C_21_H_20_O_11_ + H]^+^ and two main fragment ions at *m*/*z* 287.10 [C_15_H_10_O_6_ + H]^+^ and *m*/*z* 161.12 [M-C_15_H_14_O_6_ + H]^+^, can be identified as astragalin. Peak 14 (t_R_ = 20.107) presenting the adduct ion at *m*/*z* 508.43 [M + H]^+^ can be ascribed to C_23_H_24_O_13_. Moreover, it produced two fragment ions at *m*/*z* 355.21 [M-glc + H]^+^ and *m*/*z* 153.02 (loss of glucoside). According to the MS/MS fragmentation information and the retention time of the standard, the compound was identified as syringetin-3-*O*-glucoside. Peak 15 (t_R_ = 21.101, *m*/*z* 273.07 [C_15_H_12_O_5_ + H]^+^) was identified as naringenin. Peak 16 was temporarily inferred as piceatannol by analyzing the parent ion *m*/*z* at 245.08 [C_14_H_12_O_4_ + H]^+^ and referring to related references [2,9]. Peaks 17 and 19 can be preliminary inferred as flavonoids compounds by analyzing their typical UV–vis spectral characteristics (λ_max_ at 254 and 350 nm). Peak 20 can be preliminary determined as kaempferol glycoside owing to its MS^2^ fragment ion at *m*/*z* at 287.04 [C_15_H_10_O_6_ + H]^+^ (loss of kaempferol aglycone). It can be observed from Table 3 that the free phenolics fractions had a wider range of phenolic compositions than bound phenolics fractions. For free phenolic fractions, FM-RTF also showed higher individual phenolic contents than UM-RTF. For FM-RTF, the highest content of astragalin (307.92 ± 5.00 μg/mL) was found in free phenolic fractions. Regardless of UM-RTF or FM-RTF, gallic acid, *p*-hydroxycinnamic acid, ellagic acid and astragalin were dominant phenolic compounds in free phenolic fraction. In contrast, gallic acid and syringetin-3-*O*-glucoside were dominant phenolic compounds in bound phenolic fraction. For UM-RTF and FM-RTF, the content of gallic acid reached to 717.24 ± 30.95 μg/mL and 507.18 ± 27.07 μg/mL in bound phenolic fraction. Wang et al. (2022ab) found high levels of gallic acid, protocatechuic acid, ellagic acid and astragalin in RTF extracts, which agreed with our study [5,6]. Zhao et al. [2] revealed that gallic acid, ellagic acid, astragalin, piceatannol, and resveratrol were the main phenolic compounds in RTF. So far, there are few studies on free and bound phenolics of RTF.

### 3.3. Antioxidant Activity

Natural antioxidants are becoming increasingly important due to their capability of preventing the oxidation of other molecules and blocking the formation of free radicals [33]. In this study, the antioxidant capacity of the extracts was evaluated by four well-known chemical assays, including free radical scavenging activities of DPPH, ABTS^+^, OH^−^ and ferric reducing antioxidant activity (FRAP) (Table 4). It was found that all sample extracts showed the antioxidant activities in a concentration dependent manner. In addition, the total antioxidant activities of FM-RTF (DPPH: 345.36 ± 5.89 μmol TE/g DM; ABTS^+^: 861.01 ± 28.55; OH^−^ μmol TE/g DM: 236.10 ± 3.92 μmol TE/g DM; FRAP: 152.15 ± 6.74 mM Fe(II)E/g DM) were significantly higher than that of UM-RTF (DPPH: 267.02 ± 13.58 μmol TE/g DM; ABTS^+^: 695.49 ± 15.02 μmol TE/g DM; OH^−^: 143.64 ± 8.26 μmol TE/g DM; FRAP: 139.04 ± 5.21 mM Fe(II)E/g DM). In spite of free or bound phenolics fraction, FM-RTF showed stronger antioxidant activity than UM-RTF. For UM-RTF, the antioxidant activities of bound phenolics (DPPH: 155.89 ± 8.54 μmol TE/g DM; ABTS^+^: 352.40 ± 8.89 μmol TE/g DM; OH^−^: 83.82 ± 5.44 μmol TE/g DM; FRAP: 72.36 ± 2.60 mM Fe(II)E/g DM) were significantly stronger than those of free phenolics (DPPH: 96.12 ± 3.48 μmol TE/g DM; ABTS^+^: 317.70 ± 3.40 μmol TE/g DM; OH^−^: 59.82 ± 2.82 μmol TE/g DM; FRAP: 64.68 ± 2.61 mM Fe(II)E/g DM). For FM-RTF, only ABTS^+^ value of free phenolics was higher that of bound phenolics, but DPPH, OH^−^, FRAP values of free phenolics were slightly lower those of bound phenolics. The correlation coefficient showed that phenolic/flavonoid compounds remarkably contributed to the antioxidant activities of RTF extracts (Figure S2). In addition, it can be observed that protocatechuic acid, *p*-hydroxycinnamic acid, luteolin-7-*O*-glucoside, and ferulic acid were positively related with the DPPH, ABTS^+^, and OH^−^ (*r* > 0.50, *p* < 0.05). Remarkably, protocatechuic acid, *p*-hydroxycinnamic acid, ferulic acid, astragalin and syringetin-3-*O*-glucoside were positively correlated with the FRAP (*r* > 0.40, *p* < 0.05). Zhao et al. (2017) reported that the antioxidant activities of *Rhodomyrtus tomentosa* (Ait.) Hassk berries extracts were significantly related with the phenolic/flavonoid contents [4]. Hamid et al. [34] also found that RTF extracts with higher TPC/TFC exhibited higher antioxidant and anti-proliferative activities.

### 3.4. Digestive Enzymes Inhibitory Activity

**Figure 2:**
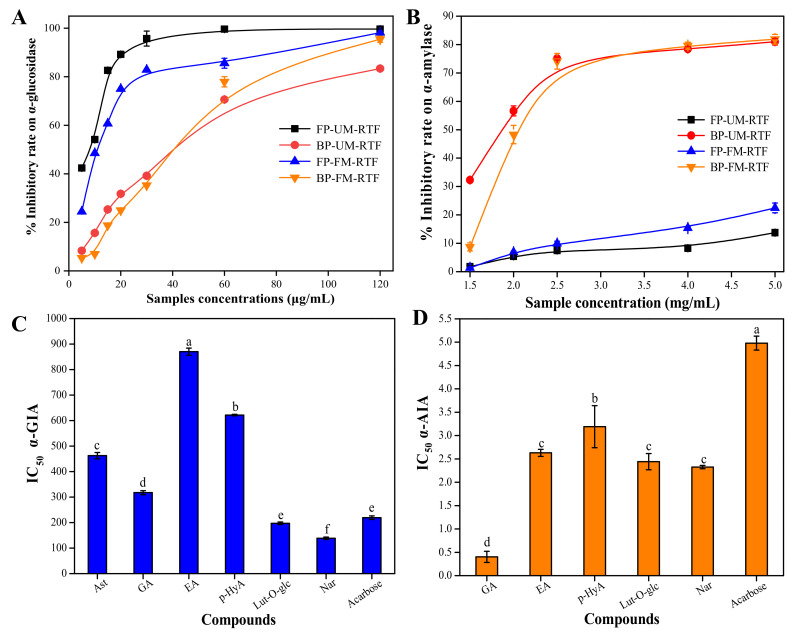
The inhibitory activities of different extracts fractions in RTF on digestive enzymes: α-glucosidase (**A**) and α-amylase (**B**). (**C**,**D**) shows the IC_50_ values of the major phenolics in RTF and acarbose against α-glucosidase/α-amylase. One-way ANOVA was used to evaluate the statistical analysis. Different lowercase letters (a–f) mean statistically significant difference (*p* < 0.05) in α-glucosidase/α-amylase inhibitory activity of the major compounds. FP-UM-RTF, free phenolic fraction of un-fully mature *R. ulmifolius* fruits; BP-UM-RTF, bound phenolic fraction of un-fully mature *R. ulmifolius* fruits; FP-FM-RTF, free phenolic fraction of fully mature *R. ulmifolius* fruits; BP-FM-RTF, bound phenolic fraction of fully mature *R. ulmifolius* fruits; Ast, astragalin; GA, gallic acid; EA, ellagic acid; *p*-HyA, *p*-hydroxycinnamic acid; Lut-*O*-glc, luteolin-7-*O*-glucoside; Nar, naringenin; α-GIA, α-glucosidase inhibitory activity; α-AIA, α-amylase inhibitory activity.

Figure 2A,B show the digestive enzymes inhibition activities of the phenolic extracts of UM-RTF and FM-RTF. It can be seen that all sample extracts exhibited the digestive enzymes inhibition activity in a concentration dependent manner. The IC_50_ values of α-glucosidase for FP-UM-RTF, BP-UM-RTF, FP-FM-RTF and BP-FM-RTF were found to be 7.02 ± 0.92 μg/mL, 25.26 ± 0.31 μg/mL, 4.95 ± 0.17 μg/mL and 13.27 ± 0.04 μg/mL, respectively. FM-RTF showed stronger α-glucosidase inhibitory activity than UM-RTF. The IC_50_ values of FP-UM-RTF, BP-UM-RTF, FP-FM-RTF and BP-FM-RTF were 39.58 ± 2.11 mg/mL, 1.04 ± 0.03 mg/mL, 18.42 ± 2.36 mg/mL and 2.19 ± 0.06 mg/Ml, respectively. It can be observed that free phenolics fractions in UM-RTF or FM-RTF showed stronger α-glucosidase inhibition activity than bound phenolic fractions. However, the bound phenolic fractions indicated stronger α-amylase inhibition activity than free phenolics fractions. In spite of UM-RTF or FM-RTF, the IC_50_ values of α-glucosidase for free and bound phenolics were significantly lower than the positive drug acarbose (IC_50_ = 219.17 ± 7.16 μg/mL), indicating the free and bound phenolics have the excellent α-glucosidase inhibition activity. However, the bound phenolics exhibited the highest α-amylase inhibition activity. Figure 2C,D show IC_50_ values of α-glucosidase and α-amylase inhibition activities of the major phenolic compounds. Of the main phenolic compounds, naringenin exhibited strongest α-glucosidase inhibitory activity (IC_50_ = 138.65 ± 4.76 μg/mL), followed by luteolin-7-*O*-glucoside (IC_50_ = 197.31 ± 5.17 μg/mL), gallic acid (IC_50_ = 317.35 ± 7.98 μg/mL) and astragalin (IC_50_ = 462.35 ± 12.27 μg/mL). *p*-Hydroxycinnamic acid (IC_50_ = 621.75 ± 2.75 μg/mL) and ellagic acid (IC_50_ = 870.21 ± 13.79 μg/mL) showed the lowest α-glucosidase inhibitory activity. For α-amylase inhibitory activity, gallic acid showed the strongest inhibitory activity (IC_50_ = 0.402 ± 0.12 mg/mL), followed by naringenin (IC_50_ = 2.325 ± 0.035 mg/mL), luteolin-7-*O*-glucoside (IC_50_ = 2.44 ± 0.17 mg/mL), ellagic acid (IC_50_ = 2.63 ± 0.075 mg/mL) and *p*-hydroxycinnamic acid (IC_50_ = 3.19 ± 0.45 mg/mL). In addition, bound phenolic fractions showed stronger α-amylase inhibitory activity than free phenolic fractions, which may be due to the fact that bound phenolic fractions had high content of gallic acid. Astragalin showed no α-amylase inhibitory activity [35]. As shown in Figure S2, TPC/TFC was closely correlated with digestive enzymes inhibitory activity (α-GIA vs. TPC, *r* = 0.781, *p* < 0.05; α-GIA vs. TFC, *r* = 0.781, *p* < 0.05; α-AIA vs. TPC, *r* = 0.854, *p* < 0.01; α-AIA vs. TFC, *r* = 0.854, *p* < 0.01). With regard to individual phenolic compounds, naringenin, luteolin-7-*O*-glucoside, gallic acid, ellagic acid and *p*-hydroxycinnamic acid were positively correlated with the α-GIA (*r* > 0.80, *p* < 0.05) and α-AIA (*r* > 0.85, *p* < 0.01). It has been found that phenolic-rich extracts from leaf-tea, edible fruits and natural products have excellent digestive enzyme inhibitory ability [35,36]. In addition, gallic acid, naringenin, luteolin-7-*O*-glucoside and ellagic acid have been confirmed to have anti-hypoglycemic effect owing to their strong inhibitory activity on digestive enzymes [37,38,39]. In short, our results verify that free and bound phenolics of RTF can be considered as a good resource of digestive enzymes inhibitors to manage postprandial blood glucose level.

### 3.5. Molecular Docking Analysis

**Figure 3:**
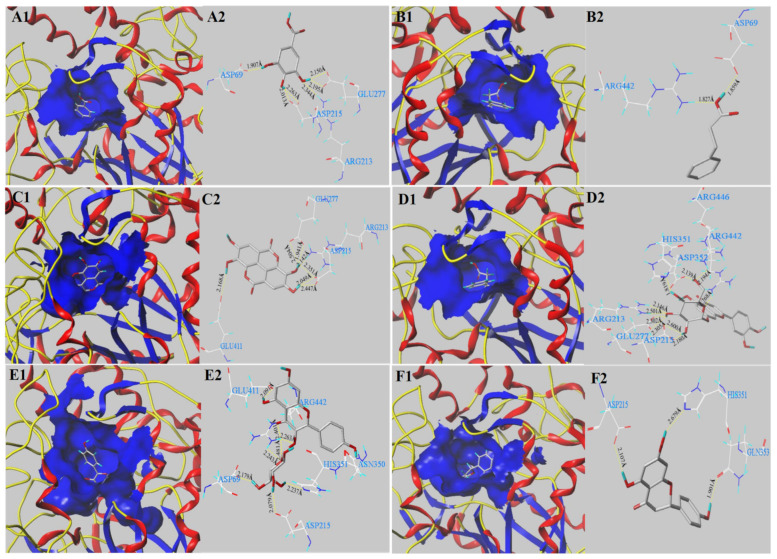
The 3D conformations of the major phenolics docked with α-glucosidase: gallic acid (**A1**,**A2**); p-hydroxycinnamic acid (**B1**,**B2**); ellagic acid (**C1**,**C2**); luteolin-7-O-glucoside (**D1**,**D2**); astragalin (**E1**,**E2**); naringenin (**F1**,**F2**) with the active pocket of α-glucosidase. The dashed yellow line presents hydrogen bonds formation.

**Figure 4:**
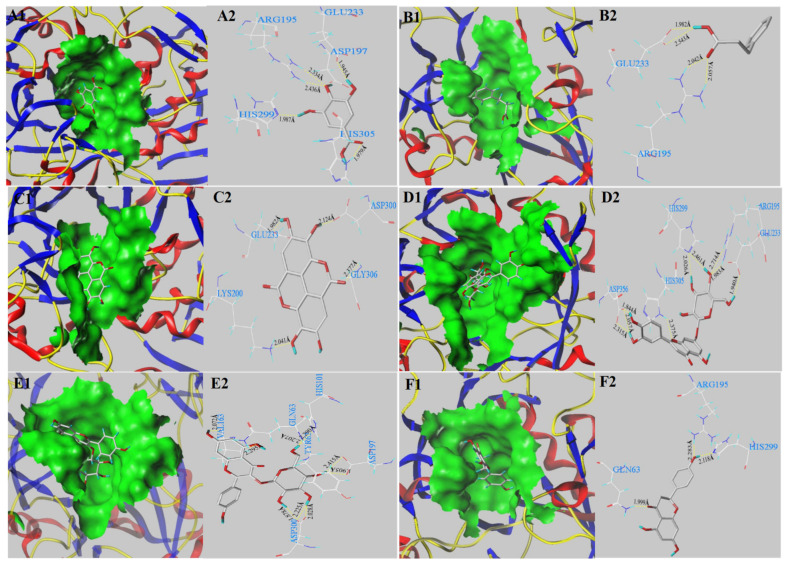
The 3D conformations of the major phenolics docked with α-amylase: gallic acid (**A1**,**A2**); *p*-hydroxycinnamic acid (**B1**,**B2**); ellagic acid (**C1**,**C2**); luteolin-7-*O*-glucoside (**D1**,**D2**); astragalin (**E1**,**E2**); naringenin (**F1**,**F2**) with the active pocket of α-amylase. The dashed yellow line presents hydrogen bonds formation.

To further illuminate inhibitory mechanisms of the main phenolics (gallic acid, *p*-hydroxycinnamic acid, astragalin, ellagic acid, naringenin and luteolin-7-*O*-glucoside) on digestive enzymes, molecular docking analysis was conducted using SYBYL-X 2.0 software. The docking results are shown in Figure 3 and Table 5. In general, C-score ≥ 4 indicates reliable docking values. T-score shows a weighted sum score of non-linear functions involving the forces between the ligands docked with the exposed receptors [26,27]. Among the investigated phenolics, it can be observed that only *p*-hydroxycinnamic acid docking with α-glucosidase and astragalin docking with α-amylase had C-scores < 4. Gallic acid (T-score of 4.49) formed six hydrogen bonds (distances ranged from 1.907 to 2.344 Å) with the active pocket of α-glucosidase (ASP 69, ASP 215, ARG 213 and GLU 277) (Figure 3(A1,A2) and Table 5). *p*-Hydroxycinnamic acid (T-score of 4.79) formed two H-bonds within 4 Å (distances of 1.654 Å–2.710 Å) with ASP 69 and ARG 442 of α-glucosidase (Figure 3(B1,B2) and Table 5). Owing to the low C-score value (<4), the result of *p*-hydroxycinnamic acid docked with α-glucosidase is unreliable. Ten H-bonds with the active pocket of α-glucosidase (ASP 215, ASP 352, ARG 213, ARG 442, ARG 446, GLU 277 and HIS 351) were formed for luteolin-7-O-glucoside, with a high docking T-score of 7.83 (Figure 3(D1,D2) and Table 5). The H-bond distance was between 1.768 Å and 2.220 Å. Astragalin, with a docking T-score of 7.48, formed eight H-bond interactions (distances of 2.091 Å–2.481 Å) with six amino acid residues (ASP 69, ASP 215, ASP 350, ARG 442, GLU 411 and HIS 351) of α-glucosidase (Figure 3(E1,E2) and Table 5). Naringenin (a docking T-score of 4.54) can interact with α-glucosidase by forming three H-bonds with three amino acid residues (ASP 215, GLN 353 and HIS 351) of α-glucosidase receptor (Figure 3(F1,F2) and Table 5). From Table 5, it can be seen that acarbose had the highest docking T-score value of 11.45. It formed thirteen H-bonds with ten binding residues of ASP 69, ASP 215, ASP 352, ARG 442, GLN 279, GLN 353, GLU 277, GLU 411, HIS 280 and TYR 158 [26,40]. These docking amino acid residues have also been reported as α-glucosidase interaction residues [26,27,30,41,42]. With regard to α-amylase, gallic acid formed five hydrogen bonds (distances of 1.907 to 2.344 Å) with ASP 197, ARG 195, GLU 233, HIS 299 and HIS 305 (Figure 4(A1,A2) and Table 5). *p*-Hydroxycinnamic acid, indicating a T-score of 4.88, could interact with α-amylase by forming four H-bonds with two amino acid residues of ARG 195 and GLU 233 (Figure 4(B1,B2) and Table 5). Ellagic acid had the lowest T-score value of 3.30. It formed four hydrogen bonds (distances of 1.907 to 2.344 Å) with the residues (ASP 300, GLY 306, GLU 233 and GLY 306) of α-amylase (Figure 4(C1,C2) and Table 5). Luteolin-7-*O*-glucoside, showing a high T-score value of 8.29, could interact with α-amylase by forming nine H-bonds with ASP 356, ARG 195, GLU 233, HIS 299 and HIS 305 (Figure 4(D1,D2) and Table 5). Astragalin (T-score value of 8.53) could interact with α-amylase by forming nine H-bonds within 4 Å (distances of 1.878 Å–2.415 Å) with ASP 197, ASP 300, GLN 63, TYR 62, HIS 101 and VAL 163 (Figure 4(E1,E2) and Table 5). However, the result of astragalin docked with α-amylase is untrustworthy because of its low C-score value. Naringenin (docking T-score of 4.19) could interact with α-amylase by forming three H-bonds with GLN 63, ARG 195 and HIS 299 (Figure 4(F1,F2) and Table 5). Acarbose, indicating a high docking T-score of 7.07, formed eleven H-bond interactions with ASP 300, GLN 63, GLU 240, GLY 306, TYR 151, LYS 200 and HIS 305 [38,40].

The molecular docking study results confirmed that the T-score value, H-bond and binding residues numbers of the compounds interacted with digestive enzymes have important effects on digestive enzyme inhibitory ability. Cai et al. (2021) confirmed that the H-bonds and active residues numbers formed by active molecules and enzymes remarkably affected the inhibitory activities on α-glucosidase or α-amlyase [40]. In this study, when the major phenolics and acarbose were docked with α-glucosidase, the numbers of formed H-bonds can be ranked as acarbose (13) > luteolin-7-*O*-glucoside (10) > astragalin (8) > ellagic acid (7) > gallic acid (6) > naringenin (3). The numbers of formed residues were in the order as acarbose (10) > luteolin-7-*O*-glucoside (7) > astragalin (6) > gallic acid (4) = ellagic acid (4) > naringenin (3). When they were docked with α-amylase, the numbers of formed H-bonds were ordered as follows: acarbose (11) > luteolin-7-*O*-glucoside (9) > gallic acid (5) > ellagic acid (4) = *p*-hydroxycinnamic acid (4) > naringenin (3). The numbers of formed residues were in the order: acarbose (7) > luteolin-7-*O*-glucoside (5) = gallic acid (5) > ellagic acid (4) > naringenin (3) > *p*-hydroxycinnamic acid (2). Moreover, gallic acid, luteolin-7-*O*-glucoside and astragalin with a high T-score value and a large number of H-bonds showed the strong inhibitory activity on α-glucosidase and α-amlyase. Acarbose, with the highest T-score, showed excellent inhibition activity on α-glucosidase. Ellagic acid and *p*-hydroxycinnamic acid with low docking T-score value exhibited the poor inhibitory activity on α-glucosidase and α-amlyase. Astragalin with high T-score exhibited good α-glucosidase inhibitory activity but poor α-amlyase inhibitory activity. In this work, gallic acid exhibited good inhibitory activity on α-glucosidase and α-amlyase, which verified that bound phenolics in RTF with high gallic acid content had stronger α-amlyase inhibitory activity than free phenolics. In addition, Priscilla et al. (2014) found that naringenin can regulate the postprandial hyperglycemia in diabetic rats by inhibiting α-glucosidase activity [38]. Li et al. (2022) observed that luteolin and luteolin-7-*O*-glucoside exhibited strong inhibition capacity on α-glucosidase [43]. Many researchers have confirmed that gallic acid can regulate blood sugar levels in db/db mice by improving glucose transporters and insulin sensitivity via PPAR-γ and Akt signaling [44]. In short, molecular docking study has revealed gallic acid, luteolin-7-*O*-glucoside, naringenin and astragalin in RTF can contribute to the inhibition activities on α-glucosidase and α-amylase, which is in agreement with the correlation co-efficient analysis results.

## 4. Conclusions

In this work, the nutrition characteristics, free and bound phenolic profiles, antioxidant properties in vitro and digestive enzymes inhibitory activities of RTF in both maturation stages were evaluated for the first time. Compared with UM-RTF, FM-RTF had higher contents of energy, ascorbic acid, organic acids and total phenolics than UM-RTF but lower TFC. In addition, gallic acid, ellagic acid and astragalin were the predominant free phenolics, while gallic acid and syringetin-3-*O*-glucoside were dominant in bound phenolic fractions. Regardless of UM-RTF or FM-RTF, stronger antioxidant activities and α-glucosidase inhibition ability were observed in free/bound phenolic fractions, while bound phenolic fractions showed stronger α-amylase inhibitory effect than free phenolics fractions. Strong correlations can be found between the phytochemical compositions and the bio-activities investigated. Furthermore, the interactions between the main phenolics in RTF and α-glucosidase/α-amylase were also analyzed by molecular docking. This study highlighted the potential value of unexploited *R tomentosa* fruits in two ripening stages, thus promoting its development and application in the food industry.

## Figures and Tables

**Table 1:** Proximate composition, free sugars, organic acids, amino acids compositions and phenolic contents of *R. tomentosa* fruit in two ripening stages. One-way ANOVA was used to evaluate the statistical analysis.

Parameters	UM-RTF	FM-RTF	*p*-Value
Moisture (%)	23.78 ± 2.16	32.43 ± 1.07	0.047
Protein (mg/g DW)	40.15 ± 0.35	38.78 ± 0.23	0.181
Ash	19.87 ± 0.78	16.95 ± 0.37	0.001
Ascorbic acid (mg/g DW)	2.13 ± 0.17	2.57 ± 0.23	0.012
Carbohydrates	51.57 ± 0.12	65.29 ± 0.33	0.04
Energy	74.32 ± 0.91	81.23 ± 0.51	0.030
Organic acids (μg/g DW)			
Oxalic acid	199.59 ± 7.23	335.14 ± 3.65	<0.001
Galacturonic acid	50.82 ± 1.63	42.69 ± 3.61	0.057
Pyruvic acid	209.25 ± 22.85	211.92 ± 15.15	0.621
Malic acid	681.92 ± 23.00	829.58 ± 7.62	<0.001
Acetic acid	378.72 ± 12.65	389.06 ± 30.57	0.051
Succinic acid	189.46 ± 29.82	468.01 ± 43.35	<0.001
Fumaric acid	1.59 ± 0.52	2.26 ± 0.36	0.023
Glucuronic acid	0.91 ± 0.06	0.13 ± 0.01	0.010
Total	1707.64 ± 95.19	2273.84 ± 26.70	0.002
Free sugars (mg/g DW)			
Glucose	124.55 ± 1.79	137.13 ± 4.96	0.043
Galactose	6.52 ± 0.46	4.17 ± 0.62	0.032
Xylose	1.58 ± 0.11	0.90 ± 0.15	0.012
Mannose	1.06 ± 0.07	1.20 ± 0.08	0.321
Arabinose	3.39 ± 0.22	2.16 ± 0.01	0.047
Rhamnose	1.01 ± 0.06	0.77 ± 0.05	0.032
Total	143.62 ± 3.24	145.88 ± 4.95	0.270
Amino acids (mg/g DW)			
Aspartic acid	0.97 ± 0.05	0.90 ± 0.06	1.23
Glutamic acid	2.21 ± 0.06	2.03 ± 0.06	0.09
Serine	0.75 ± 0.05	0.72 ± 0.01	1.02
Histidine	0.92 ± 0.02	0.88 ± 0.01	0.67
Glycine	1.33 ± 0.07	1.20 ± 0.06	0.73
Threonine	0.79 ± 0.02	0.74 ± 0.01	0.24
Arginine	1.76 ± 0.00	1.71 ± 0.00	0.17
Alanine	0.94 ± 0.01	0.92 ± 0.02	0.16
Tyrosine	1.18 ± 0.03	1.09 ± 0.00	0.12
Valine	0.88 ± 0.05	0.86 ± 0.02	0.52
Methionine	0.65 ± 0.00	0.62 ± 0.00	0.98
Phenylalanine	0.85 ± 0.05	0.70 ± 0.03	0.08
Isoleucine	0.81 ± 0.03	0.83 ± 0.01	0.09
Leucine	1.40 ± 0.05	1.26 ± 0.04	0.13
Lysine	0.76 ± 0.02	0.77 ± 0.03	0.08
Total	16.21 ± 0.05	15.27 ± 0.36	0.042
Phenolics content (mg GAE/g DW)			
Free phenolic	11.20 ± 0.23	12.43 ± 0.16	0.041
Bound phenolic	11.23 ± 1.20	17.38 ± 1.18	0.004
Total	24.01 ± 1.52	31.53 ± 1.36	0.007
Flavonoids content (mg RE/g DW)			
Free flavonoid	5.32 ± 0.05	4.35 ± 0.04	0.061
Bound flavonoid	3.17 ± 0.10	2.73 ± 0.08	0.032
Total	9.87 ± 0.16	7.74 ± 0.15	0.038

**Table 2:** Identification of free and bound phenolic fractions in RTF by HPLC-ESI-qTOF-MS/MS.

Peak No.	RT (min)	λ_max_ (nm)	[M + H]^+^	MS/MS (*m*/*z*)	Mw	Formula	Compounds	Error	Reference
1	3.015	245, 278	171.02	171.02, 127.02	170	C_7_H_6_O_5_	Gallic acid	0.06	Standard, MS/MS
2	3.672	260, 281	153.02	153.02, 108.02	154	C_7_H_6_O_4_	Protocatechuic acid	0.32	Standard, MS/MS
3	9.071	254, 350	169.15	169.15	168	C_8_H_8_O_4_	Vanillic acid	0.15	Standard, MS/MS
4	9.982	261, 280	165.14	165.14, 137.02	165	C_9_H_8_O_3_	*p*-Hydroxycinnamic acid	−0.38	Standard, MS/MS
5	10.208	256, 350	303.05	303.05, 259.01, 193.12	302	C_14_H_6_O_8_	Ellagic acid	0.58	Standard, MS/MS
6	13.727	254, 350	463.12	463.12, 302.03, 162.10, 151.05	462	C_22_H_22_O_11_	Peonidin-3-*O*-*β*-galactoside	−1.31	MS/MS, [5,6]
7	14.259	257, 360	493.13	493.13, 492.13, 346.29, 332.29, 161.01, 151.02	493	C_23_H_24_O_12_znj	Tricin 5-*O*-*β*-D-glucoside	−1.26	MS/MS, [5,6]
8	14.518	254, 280	149.02	149.02, 148.01	148	C_9_H_8_O_2_	*p*-Coumaraldehyde	0.59	MS/MS
9	14.985	254, 280	174.51	174.51, 119.54	173	-	-	0.22	MS/MS
10	15.938	260, 360	449.10	449.10, 287.10, 162.02, 161.02, 151.02	448	C_21_H_20_O_11_	Luteolin-7-*O*-glucoside	−0.17	Standard, MS/MS
11	16.217	257, 350	229.08	229.08, 228.03, 185.06, 183.08, 159.08, 143.05	228	C_14_H_12_O_3_	Resveratrol	1.05	MS/MS, [32]
12	17.195	261, 280	195.05	195.05, 178.02, 134.04	194	C_10_H_10_O_4_	Ferulic acid	0.02	MS/MS
13	17.682	254, 350	449.39	449.39, 287.10, 161.12	448	C_21_H_20_O_11_	Astragalin	−0.05	Standard, MS/MS
14	20.107	280, 350	509.43	508.43, 355.21, 161.01, 153.02	508	C_23_H_24_O_13_	Syringetin-3-*O*-glucoside	0.23	Standard, MS/MS
15	21.101	257, 350	273.07	273.07, 151.08	272	C_15_H_12_O_5_	Naringenin	−1.22	Standard, MS/MS
16	22.125	254, 280	245.08	245.08, 245.02	244	C_14_H_12_O_4_	Piceatannol	0.30	MS/MS, [2,9]
17	26.892	254, 350	287.06	287.06, 286.05	286	C_15_H_12_O_6_	-	−0.53	MS/MS
18	27.254	254, 350	287.04	287.05, 286.04	286	C_15_ H_10_ O_6_	Fisetin	2.10	MS/MS
19	27.951	254, 350	449.11	449.11, 287.10, 153.10	448	C_21_ H_20_ O_11_	-	3.95	MS/MS
20	29.078	254, 350	634.19	634.19, 287.04, 161.05, 153.10	634	C_30_H_34_O_15_	Kaempferol glycoside	1.13	MS/MS

**Table 3:** The contents of individual phenolic compounds of RTF in two ripening stages.

Phenolic Compounds (μg/g DW)	UM-RTF		FM-RTF	
	Free Phenolic (FP)	Bound Phenolic (BP)	Free Phenolic (FP)	Bound Phenolic (BP)
Gallic acid	70.24 ± 1.57 Aa	717.24 ± 30.95 Bd	82.72 ± 5.56 Ab	507.18 ± 27.07 Bc
Protocatechuic acid	2.13 ± 0.62 Aa	3.57 ± 0.21 Bb	6.42 ± 0.62 Bc	2.49 ± 0.21 Aa
Vanillic acid	N.D.	7.12 ± 0.56	N.D.	N.D.
*p*-Hydroxycinnamic acid	20.17 ± 0.99 Bc	3.60 ± 0.11 Aa	17.53 ± 1.48 Bb	3.09 ± 0.22 Aa
Ellagic acid	38.16 ± 1.00 Bc	6.41 ± 0.70 Aa	55.30 ± 6.03 Bd	7.43 ± 4.68 Ab
Luteolin-7-*O*-glucoside	9.91 ± 0.20 Bc	6.08 ± 0.25 Ab	28.94 ± 5.82 Bd	4.99 ± 0.27 Aa
Ferulic acid	0.57 ± 0.06 Aa	1.58 ± 0.05 Bb	5.82 ± 1.28 Ad	4.85 ± 0.15 Ac
Astragalin	59.73 ± 5.26 a	N.D.	307.92 ± 5.00 b	N.D.
Syringetin-3-*O*-glucoside	9.29 ± 0.16 Aa	11.17 ± 0.58 Bb	N.D.	10.83 ± 0.29 b
Naringenin	12.62 ± 0.29 a	N.D.	11.82 ± 0.57 a	N.D.

Notes: Two-way ANOVA was used to evaluate the statistical analysis. Different uppercase letters (A, B) at the same row mean statistically significant differences in FP and BP at the same ripening stages (*p* < 0.05). Different lowercase letters (a–d) at the same row mean statistically significant differences in UM-RTF and FM-RTF (*p* < 0.05). N.D. Not detected; UM-RTF, un-fully mature *R. ulmifolius* fruits; FM-RTF, fully mature *R. ulmifolius* fruits; FP, free phenolic; BP, bound phenolic.

**Table 4:** Antioxidant activities and digestive enzyme inhibitory activity (IC_50_) of *R. tomentosa* fruit in two ripening stages.

Stages	UM-RTF	FM-RTF	*p* Value
DPPH (μmol TE/g DW)			
FP	96.12 ± 3.48 Aa	179.77 ± 3.58 Bb	<0.001
BP	155.89 ± 8.54 Ba	152.43 ± 0.08 Aa	0.382
Sum	267.02 ± 13.58 a	345.36 ± 5.89 b	0.004
ABTS^+^ (μmol TE/g DW)			
FP	317.70 ± 3.40 Aa	395.50 ± 15.46 Ab	0.041
BP	352.40 ± 8.89 Ba	435.34 ± 12.39 Bb	0.002
Sum	695.49 ± 15.02 a	861.01 ± 28.55 b	<0.001
OH^−^ (μmol TE/g DW)			
FP	59.82 ± 2.82 Aa	144.32 ± 1.69 Bb	<0.001
BP	83.82 ± 5.44 Ba	91.78 ± 2.23 Ab	0.021
Sum	143.64 ± 8.26 a	236.10 ± 3.92 b	0.005
FRAP (mM Fe(II)E/g DW)			
FP	64.68 ± 2.61 Aa	76.84 ± 4.38 Ab	0.042
BP	72.36 ± 2.60 Ba	75.31 ± 1.36 Aa	0.075
Sum	139.04 ± 5.21 a	152.15 ± 6.74 b	0.036
Anti-α-glucosidase activity (IC_50_, μg/mL)			
FP	7.02 ± 0.92 Ab	4.95 ± 0.17 Aa	0.021
BP	25.26 ± 0.31 Bb	13.27 ± 0.04 Ba	<0.001
Anti-α-amylase activity (IC_50_, mg/mL)			
FP	39.58 ± 2.11 Bb	18.42 ± 2.36 Ba	<0.001
BP	1.04 ± 0.03 Aa	2.19 ± 0.06 Ab	0.038

Notes: Two-way ANOVA was used to evaluate the statistical analysis. Different uppercase letters (A, B) at the same column mean statistically significant differences in FP and BP (*p* < 0.05). Different lowercase letters (a, b) at the same row mean statistically significant differences in UM-RTF and FM-RTF (*p* < 0.05). FP, free phenolic; BP, bound phenolic; UM-RTF, un-fully mature *R. ulmifolius* fruits; FM-RTF, fully mature *R. ulmifolius* fruits.

**Table 5:** The analysis results of the major phenolic compounds and controls docking with *α*-glucosidase or *α*-amylase.

Enzymes	Major Phenolics	C-Score	T-Score	*n* (Binding Residues)	*n* (H-Bond Formation)	Active Amino Acid Residues
*α*-Glucosidase	Gallic acid	4	4.49	4	6	ASP 69, ASP 215, ARG 213, GLU 277
	*p*-Hydroxycinnamic acid	3	4.79	2	2	ASP 69, ARG 442
	Ellagic acid	4	2.79	4	7	ASP 215, ARG 213, GLU 277, GLU 411
	Luteolin-7-*O*-glucoside	5	7.83	7	10	ASP 215, ASP 352, ARG 213, ARG 442, ARG 446, GLU 277, HIS 351
	Astragalin	5	7.48	6	8	ASP 69, ASP 215, ASP 350, ARG 442, GLU 411, HIS 351
	Naringenin	4	4.54	3	3	ASP 215, GLN 353, HIS 351
	Acarbose	5	11.45	10	13	ASP 69, ASP 215, ASP 352, ARG 442, GLN 279, GLN 353, GLU 277, GLU 411, HIS 280, TYR 158
*α*-Amylase	Gallic acid	5	4.52	5	5	ASP 197, ARG 195, GLU 233, HIS 299, HIS 305
	*p*-Hydroxycinnamic acid	5	4.88	2	4	ARG 195, GLU 233
	Ellagic acid	4	3.30	4	4	ASP 300, GLY 306, GLU 233, GLY 306
	Luteolin-7-*O*-glucoside	4	8.29	5	9	ASP 356, ARG 195, GLU 233, HIS 299, HIS 305
	Astragalin	2	8.53	6	9	ASP 197, ASP 300, GLN 63, TYR 62, HIS 101, VAL 163
	Naringenin	4	4.19	3	3	GLN 63, ARG 195, HIS 299
	Acarbose	5	7.07	7	11	ASP 300, GLN 63, GLU 240, GLY 306, TYR 151, LYS 200, HIS 305

## Data Availability

Data are contained within the article.

## Supplementary Materials

The following are available online at https://www.mdpi.com/article/10.3390/antiox11071390/s1, Figure S1: The flow diagram of extraction and analysis for *Rhodomyrtus tomentosa* fruit (RTF) in two ripening stages. Figure S2: Heatmap analysis of correlation matrix between the major compounds and the bioactivities of RTF.

## Abbreviations

RTF	*Rhodomyrtus tomentosa* fruit
UM-RTF	Un-fully mature *Rhodomyrtus tomentosa* fruit
FM-RTF	Fully mature *Rhodomyrtus tomentosa* fruit
DW	Dry weight
*α*-GIA	*α*-Glucosidase inhibitory activity
*α*-AIA	*α*-Amylase inhibitory activity
TPC	Total phenolic content
TFC	Total flavonoid content
FP	Free phenolic
BP	Bound phenolic
