# Supplementary material for: Rhodomyrtus tomentosa Fruits in Two Ripening Stages: Chemical Compositions, Antioxidant Capacity and Digestive Enzymes Inhibitory Activity
Source: Antioxidants (Basel). 2022 Jul 18;11(7):1390. doi: 10.3390/antiox11071390 (PMC9311718; doi:10.3390/antiox11071390)
Supplement: Supplementary file 1 [file antioxidants-11-01390-s001.zip › antioxidants-1811289-supplementary/Figure S1.pdf]

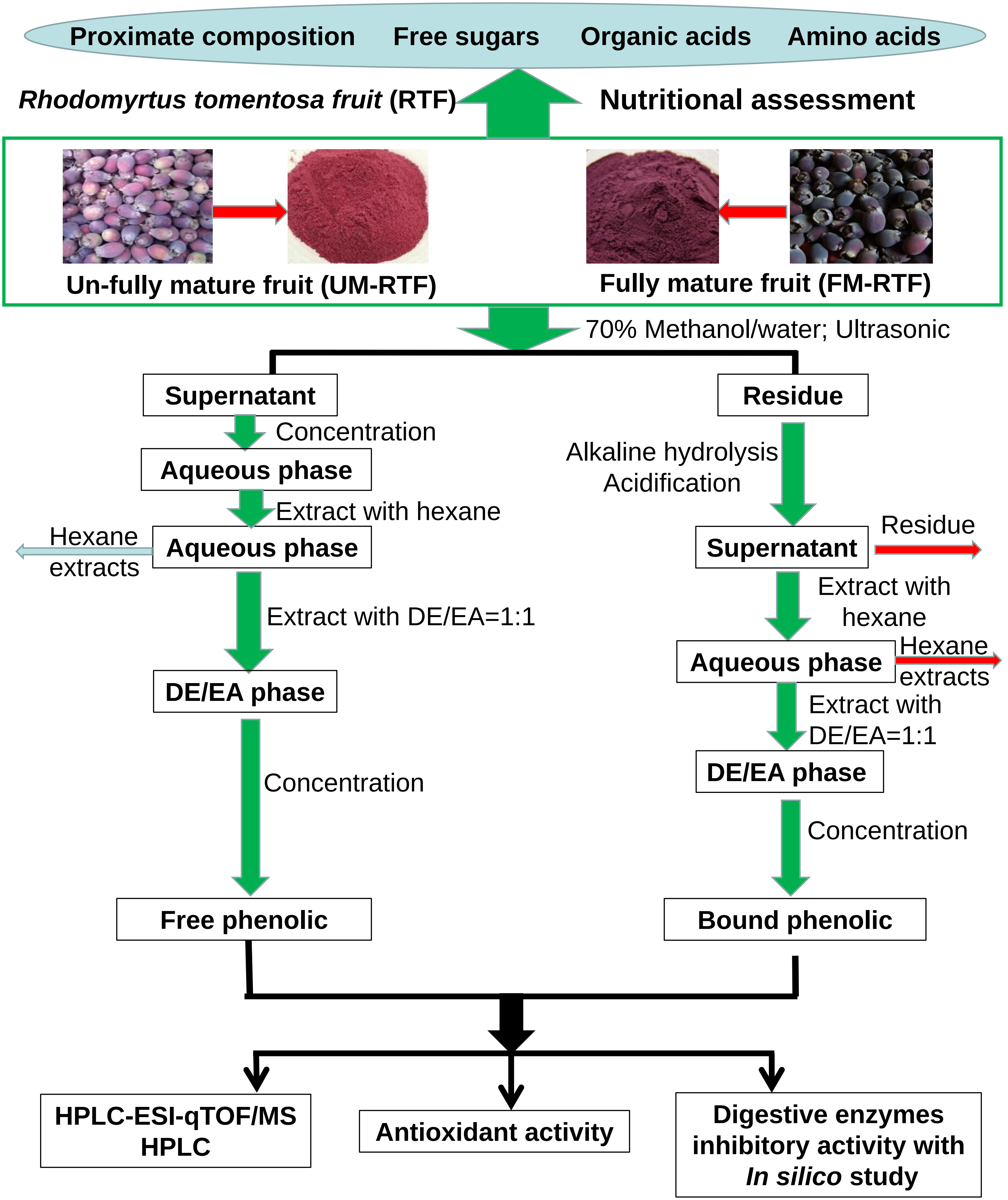
Proximate composition
Free sugars
Organic acids
Amino acids
Rhodomyrtus tomentosa fruit (RTF)
Nutritional assessment
Un-fully mature fruit (UM-RTF)
Fully mature fruit (FM-RTF)
70% Methanol/water; Ultrasonic
Supernatant
Residue
Concentration
Aqueous phase
Extract with hexane
Hexane extracts
Aqueous phase
Extract with DE/EA=1:1
DE/EA phase
Concentration
Free phenolic
Alkaline hydrolysis
Acidification
Supernatant
Residue
Extract with hexane
Aqueous phase
Hexane extracts
Extract with DE/EA=1:1
DE/EA phase
Concentration
Bound phenolic
HPLC-ESI-qTOF/MS
HPLC
Antioxidant activity
Digestive enzymes inhibitory activity with In silico study
