# Supplementary figures and images for: Rhodomyrtus tomentosa Fruits in Two Ripening Stages: Chemical Compositions, Antioxidant Capacity and Digestive Enzymes Inhibitory Activity
Source: Antioxidants (Basel). 2022 Jul 18;11(7):1390. doi: 10.3390/antiox11071390 (PMC9311718; doi:10.3390/antiox11071390)

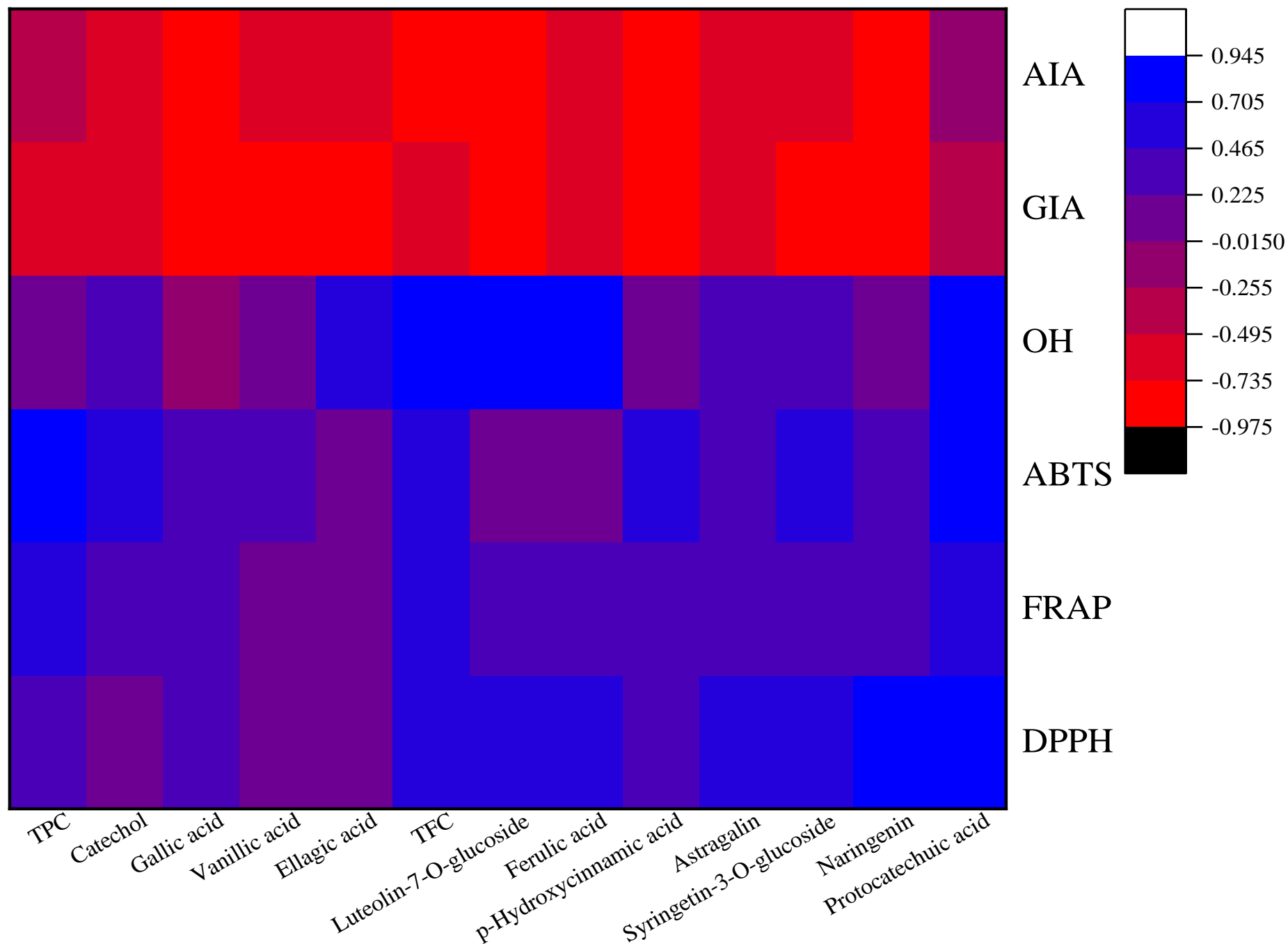

Supplement: Supplementary file 1 [file antioxidants-11-01390-s001.zip › antioxidants-1811289-supplementary/Figure S2.pdf]
